# Extensive Rearing Systems in Poultry Production: The Right Chicken for the Right Farming System. A Review of Twenty Years of Scientific Research in Perugia University, Italy

**DOI:** 10.3390/ani11051281

**Published:** 2021-04-29

**Authors:** Alessandro Dal Bosco, Simona Mattioli, Alice Cartoni Mancinelli, Elisa Cotozzolo, Cesare Castellini

**Affiliations:** Department of Agricultural, Food and Environmental Science, University of Perugia, Borgo XX Giugno, 74, 06100 Perugia, Italy; alessandro.dalbosco@unipg.it (A.D.B.); simona.mattioli@unipg.it (S.M.); elisa.cotozzolo@libero.it (E.C.); cesare.castellini@unipg.it (C.C.)

**Keywords:** poultry, extensive rearing systems, welfare, quality, sustainability, management

## Abstract

**Simple Summary:**

The aim of this review paper was to retrace the research journey of the researchers of the Department of Agricultural, Food, and Environmental Science at the University of Perugia, Italy that lasted twenty years and draw updated guidelines regarding the best synergy between chicken type and environment in extensive rearing systems in order to optimize animal welfare, quality, and environmental impact, linked with economical sustainability.

**Abstract:**

The demand for poultry meat, being cheaper than red meat, will drive worldwide production of this product. Accordingly, an increase in production up to 16% is expected in 2025, most of which will occur in developing countries. Most poultry meat production is realized with intensive production systems, and extensive rearing systems (ERS) of poultry (organic, free-range, and low-input) represent only a small portion of poultry production in the EU (about 5%). However, there is an increasing interest in such rearing systems to maintain the good image of product and environmental sustainability, improved animal welfare, and meat quality with an annual trend of growth of about 10%. The aims of this work were to summarize the activities and the viewpoint of the researchers of the Department of Agricultural, Food, and Environmental Science of the University of Perugia (Italy). One of the most important goals of the research unit was the challenge of identifying the best poultry genotypes for ERS, which are important not only for the food industry but also for the improvement of human nutrition. Only the definition of the best genotypes adapted to ERS through the measurement of a wide panel of traits—genetic, physiologic, and behavior—and not only relying on daily weight gain will allow us to achieve this goal.

## 1. Introduction

Due to the lower cost of poultry meat than red meat, its world production for 2025 is expected to grow by 16% compared to the reference period 2013–2015 (+20% compared to the previous decade) [1]. Poultry meat will increase from 115,192 kt rtc (kilo tons ready to cook) in 2016 to 131,255 kt rtc by 2025 and Argentina, Brazil, Mexico, Russia, Ukraine, and the United States will be the top producers. In Asia, the leaders will be China, India, Indonesia, Iran, and Thailand.

Most poultry meat derives from intensive poultry production systems (95%) and a small portion (5%) from the extensive rearing systems (ERS) such as organic, free-range, and low-input production systems. Obviously, these rearing systems are less standardisable than intensive ones and are not the same in every country; there are even major differences between countries within the European Union, due to climate, labor, and feed costs; availability of alternative genotypes and land resources; and willingness of consumers to pay for premium products.

Concerning the organic systems, in general, chickens must have access to an abundance of fresh air, daylight, and outdoor space. More specifically, every effort has to be made to allow chickens to live as natural a life as possible. Production is less intensive, 4 m² run space has to be available (Commission Regulation No 889/2008), and the feed requirements for organic chickens are very strict. A minimum of 20% of the feed must be produced at the farm or in the region. The grains must be produced GMO-free and are subject to stringent requirements regarding pesticide and fertilizer use. There is increasing interest in such rearing systems as they provide a good image for the product and environmental sustainability, improved animal welfare, and meat quality with an annual trend of growth of about 10% [2].

Twenty years ago, some researchers from the Department of Agricultural, Food, and Environmental Sciences of the University of Perugia, Italy, began to study the different aspects of poultry production in ERS, which resulted in the first scientific paper published on this topic (that we will call “number one” [3]). The aim of this first study was to compare the quantitative and qualitative differences between commercial and organic poultry production systems. Until then, organic production was mainly considered a strict production protocol with no or limited effect on the quality of products. This paper anticipates a new idea and approach, consisting of assessing the role of each mandatory rule (genetic strain, feed, and pasture) of rearing systems in the production performance and on meat quality. Since that time, one remark seems very timely concerning the Mediterranean areas: “It is useless to give large space to chickens that are not able to exploit it… but at the same time it is useless to use chickens capable of grazing without providing them with grass.” In fact, in some countries and markets, there was widespread use of commercial meat-type broilers in ERS, which was mainly due to their high growth performance and breast yield.

In this review, we will discuss this journey that covers twenty years of our research in Italy, drawing an updated conclusion regarding the best synergy between chicken type and environment in ERS, in order to optimize animal welfare, quality, and environmental impact with an economically feasible proposal. Some of the researchers reported were performed in organic rearing systems regulated by Commission Regulation No 889/2008, whereas those in Table S1 clarify the main differences between the used genotypes in all the trials referred to in this review.

## 2. Genetics

The first research efforts focused on poultry genotypes, considering it essential to define the chicken type that is better suited for the natural environmental conditions of ERS. Accordingly, together with productive performance, other traits such as walking activity, exploratory attitude, immune response, thermo-tolerance, and welfare play a prominent role in the adaptability of chickens to ERS.

In the highly cited (417 citations, source Scopus) “number one”, the effect of organic production on broiler carcass and meat quality was assessed while still unaware of all the problems related to this rearing system and on how many factors were linked to obtain this production. Ross 308 male chickens were assigned to two different systems of production: conventional (housing in indoor pen, 0.12 m^2^/bird) and organic (housing in an indoor pen, 0.12 m^2^/bird with access to a grassed paddock, 4 m^2^/bird). The results showed that the organic chickens had carcasses with higher breast and drumstick percentages and lower levels of abdominal fat; moreover, the muscles had lower ultimate pH and water-holding capacity. Other than cooking loss, the values for lightness, shear, Iron (Fe), Polyunsaturated Fatty Acids (PUFA) of the n-3 series, and Thiobarbituric Reactive Substances (TBARS; i.e., lipid oxidation) were found to be higher in organic chickens. The high level of lipid oxidation and the appearance of behavioral (higher frequency of lying down, lower kinetic activity) and carcass defects raised the first doubts about the real possibility of using commercial hybrids selected for completely different environments. This discrepancy between the genetic strain and the farming system prompted researchers of the University of Perugia to start research paths aimed at exploring the complex genetic, metabolic, feeding, environmental, logistical, and technological aspects associated with different production systems. All these factors are fundamental in order to find a more suitable chicken genotype for extensive farming systems.

Following *number one*, additional experiments [4] compared the performance and behavior of three different chicken strains reared under the organic rearing system described earlier. The strains had very slow (Robusta maculata), slow (Kabir), and fast (Ross 308) growth rates. Robusta maculata and Kabir chickens showed more intense walking activity and more foraging behavior, and consequently their antioxidant capacity was higher. Ross 308 chickens were confirmed with a good growth rate, carcass weight, and feed conversion index, but the mortality and culling rate were higher, demonstrating for the first time that fast growing (FG) strains did not adapt well to organic production. Robusta maculata showed lower productive performance, although the mortality was low and Kabir birds gave intermediate results. In short, even in this study, the poor adaptability of the FG strains was confirmed in terms of significantly high mortality (10, 5, and 4%, respectively, for Ross 308, Kabir, and Robusta maculata), poor grazing (Time spent outdoor 35, 60, and 65% total time, respectively, for Ross 308, Kabir, and Robusta maculata), and antioxidant capacity (522, 700, and 715 μmol HClO mL^−1^, respectively, for Ross 308, Kabir, and Robusta maculata).

Subsequent investigations were carried out to investigate the meat quality of the above-mentioned genotypes with different growth rates [5,6]. FG Ross 308, medium-growing (MG), and slow growing (SG) Kabir and Robusta maculata were reared according to EU directive on the organic production system requirements, which defines a paddock with grass pasture (4 m^2^/bird) and a slaughter age greater than 81 d. The meat quality was affected by the different degree of maturity with respect to complete somatic development of the strains at slaughter age, which was 70% for Ross 308, 52% for Kabir, and 78% for Robusta maculata. Ross 308 and Kabir were slaughtered at 81 days, whereas Robusta maculata required 120 days to reach a commercial weight (live weight > 2 kg). The meat of all three genotypes showed good qualitative traits, but that of Ross 308 chickens had more fat, lower ultimate pH and iron, and was paler. Kabir chickens, being the least mature strain, had the highest moisture content with a high cooking loss. The SG genotypes adapted well to the extensive rearing conditions, while the FG genotypes showed an ineffective muscle response to greater activity in terms of antioxidant capacity so that the oxidative stability of the meat was reduced.

In the same trials, *Pectoralis major* muscles were excised and analysed after 0, 24, 48, 72, and 96 h of storage at 4 °C under continuous fluorescent illumination (2.300 lx. In addition, in this trial, the genotype greatly affected the physiochemical characteristics and sensory evaluation. The meat from Ross 308 chickens showed high TBARS, perhaps due to selection for growth rate that reduced their adaptability to greater space allowance and to poorer environmental conditions. This high TBARS was also negatively correlated with lightness and yellowness. The results of the panel test showed that the consumer panel preferred the meat of Kabir birds until 48 h of storage, during which the level of TBARS remained below 2 mg of malondialdehyde per kg of meat. Successively, meat acceptability decreased over time and the panellists were not able to discriminate the differences.

**Figure 1:**
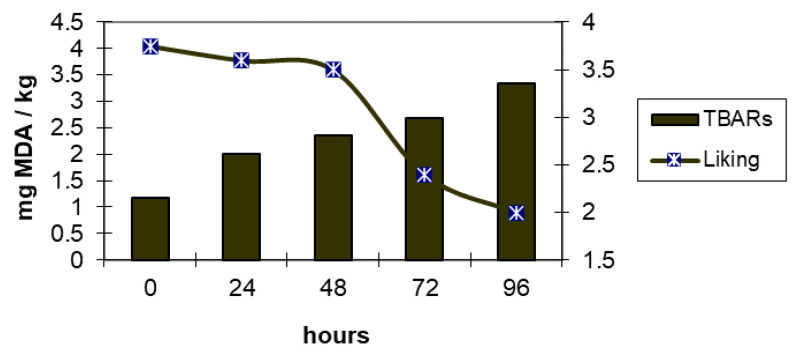
Relationship between evolution of TBARS and liking in Kabir meat (modified by [6]). MDA: Malondialdehyde; TBARS: Thiobarbituric acid reactive substances.

**Figure 2:**
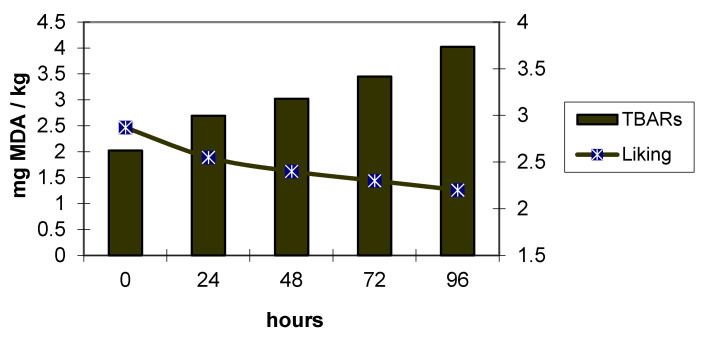
Relationship between evolution of TBARS and liking in Ross 308 meat (modified by [6]). MDA: Malondialdehyde; TBARS: Thiobarbituric acid reactive substances.

Figure 1 and Figure 2 clearly show the negative correlation (−0.82 and −0.86) between liking (mainly in terms of impact on the flavour) and oxidative processes. In Ross 308 chickens, with the worst oxidative status, the acceptability started to decrease at 24 h, when the malondialdehyde level was greater than 2.5 mg/kg of meat. In Kabir, the decrease in the acceptability was relevant starting at 72 h, with an amount of malondialdehyde analogous to that of Ross 308 at 24 h.

This first block of experiments underlined the strong correlations between genotype and the product quality obtained in poultry ERS; in particular, trials stated that ERS could allow the acquisition of leaner and tastier meat but with high oxidation and shorter shelf-life. However, the question remained, by how much?

The answer seems to always be linked to the genotype. The focus of most research is as follows: higher kinetic activity and the less controlled environmental conditions contribute to worsening of oxidative status, mainly in strains selected for fast growth and high productive performance, and the amount of TBARS shows a strong negative correlation with sensory evaluation.

At this point, researchers ask themselves, in what ways could we move forward and begin to combine the quality (and therefore adaptability; ability to exploit pasture; and rusticity) with economically acceptable production?

In 2011, we verified the feasibility of a cross between a local breed and a heavier one and, therefore, combined frugality and productivity [7]; in particular, the aim of this research was to evaluate the performance and carcass and meat quality of Ancona or crossbred chicks (Cornish × Ancona) that had been organically reared. The Cornish × Ancona crossbreed showed better productive performance than pure Ancona chicks (final live weight 2369 vs. 1874 g; feed efficiency 3.2 vs. 3.9; ready-to-cook-carcass 1622 vs. 1299 g). In regard to the breast chemical composition, strain affected only lipid content, ultimate pH, and colour. The fatty acid profile of the breast did not show significant variations, whereas TBARS was higher in Cornish × Ancona birds (1.75 vs. 1.50 mg MDA/kg). These results showed that, in respect to purebred, Cornish × Ancona chickens exhibited better performance and similar meat characteristics. Thus, it is possible to conclude that the crossbreed could represent a good compromise between economic sustainability and the safeguard of a local breed.

Local poultry breeds throughout the world are in danger of extinction due to their replacement by more productive poultry strains. However, some of these local breeds could have traits and genes relevant for adaptation to ERS (i.e., resistance to heat stress, higher immune response, kinetic activity, and some meat characteristics). The consistency of these breeds is also critical in Italy, and for this reason, it is necessary to improve their use widespread by including the local strains in preservation and in productive programs. Recently, in Italy, the Genealogic Book of poultry was developed, which includes 22 chicken breeds, and other breeds of turkey, ducks, geese, and pigeons. Different genetic studies were carried out at the University of Perugia. First, the maternal genetic origin of five Italian local chicken breeds (Ancona, Livorno, Modenese, Romagnola, and Valdarnese bianca) based on mitochondrial DNA information was investigated. Moreover, the extent of the genetic diversity, population structure, and genetic relationships among these chicken populations was assessed using 27 microsatellite markers [8]. A second study investigated the genetic diversity and relationships among 16 local breeds of chicken originating from five countries (Italy, Spain, Serbia, Albania, and the Republic of Malta) of the Mediterranean basin [9]. In addition, the genomic variability of local Italian chicken breeds was studied using single nucleotide polymorphisms (SNPs) to understand their genetic diversity and population structure, concluding that these breeds have conserved authentic genetic patterns and that these results can improve conservation strategies. Moreover, the conservation of local breeds may play an important role in the local economy as a source of high-quality products [10].

A previous study determined the consistency and characteristics of Ancona and Livorno chicken breeds in their region of origin (Central Italy [11]). The authors concluded that the diffusion of the breeds depends on the region and that the Ancona breed is most present in the Marche region, while the Livorno breed is mainly bred in Tuscany.

**Figure 3:**
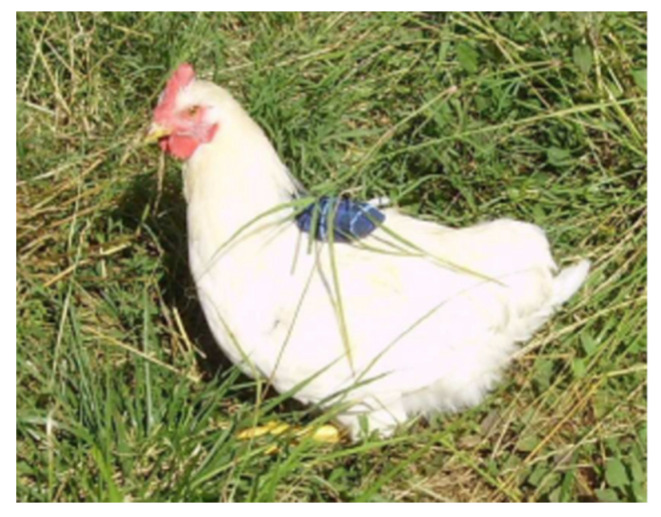
Chicken with the, Super Track stick GPS.

After underlining the importance of the genotype on chicken adaptability to ERS, we tried to show, using objective methods, the use of pasture by different strains. The idea was to use a global positioning system (GPS, Super Track stick,Atex Internationa, Route d’Esch, Luxembourg) device to evaluate the activities of different chicken genotypes on pasture [12]. Two chicken strains (100 Ancona crossbred, SG and 100 Ross 308, FG birds) were reared separately in four indoor pens, each with access to a grass paddock (10 m^2^/bird). The instrument was equipped with a USB key for quick viewing on Google Earth’s 3-D model, giving information concerning the date, hour, environmental conditions, and coordinates of monitored birds. Monitors weighed approximately 50 g and were attached to the back of the bird using a belt Velcro system (Figure 3).

The obtained results showed a clear tendency of FG birds to stay indoors instead to explore the external run and feed on the available pasture. Visual observations and GPS records agree that SG birds performed more active behaviors, stood less, and spent more time outdoors than indoors. According to the GPS tracks, SG chickens covered an average daily distance roughly 10 times higher than FG, which was for the first time objectively confirmed (Table 1).

Another interesting finding was related to footpad lesions and breast blisters. The incidence of footpad lesions was dramatically higher in FG chickens than in SG chickens. In fact, more than 70% of FG birds had the highest footpad lesion score, whereas only 1.05% of SG birds showed minimal lesions. The same was true for breast blisters, which were noticeably higher in FG birds than in SG birds (completely absent). Unfortunately, SG birds displayed extremely poor productive performance, and the use of such strains in commercial production would be unprofitable.

In another study [13], the meat characteristics of Cobb 700, Naked Neck, Kabir, and Brown Classic Lohman chickens were compared. In this trial, meat functional properties of FG and MG strains appeared to be much more attractive for both industry and consumer use (lower drip and cook losses and higher tenderness), whereas from a nutritional point of view, meat from SG ones appeared healthier (less fat and higher content of n-3 PUFA) and thus might better fit consumers’ expectations of healthier products.

Having defined the enormous differences between chickens with different growth rates and the impossibility of using commercial FG or local pure breeds for ERS, the researchers refined their experimental activities to define the most adaptable genotype. Thus, adaptability continues to be the key word, so far as it is crucial to point out all the criteria in order to define the SG genotypes and their adaptability as recommended by the article 12 of Regulation (EC) n. 889/2008. In particular, this regulation establishes that, “in organic livestock production, the choice of breeds should consider their capacity to adapt to local conditions, their vitality and their resistance to disease, and a wide biological diversity should be encouraged.” From this point of view, it is crucial to find a good compromise between welfare, adaptability to the natural environment, biodiversity, and productive performance of animals. In European countries, the choice of poultry genotypes is only based on daily weight gain, and there is no univocal and official classification to identify poultry strains adapted to ERS.

Therefore, many authors [14,15] classify poultry genotypes by relying only on productive performance as follows:

- Fast growing (FG) strains, adapted to intensive rearing systems, able to reach slaughter weight in a short time (about 2.5 kg in 40 days) with extremely high breast yield.
- Slow growing (SG) strains, which represent a heterogeneous group of chickens made up of commercial strains, selected by poultry companies for outdoor farming and by local poultry breeds.

Subsequently, the concept of medium growing, also called “slower growing” by the genetic companies, emerged to indicate a subject with intermediate characteristics, both from the point of view of production and from the ability of the birds to adapt to ERS.

Based on these considerations, the aim of this further study carried out in collaboration with the Italian Minister of Agriculture [16] was to deepen the knowledge on the ethology and well-being of eight different poultry breeds through a complex multifunctional approach that took into consideration various aspects such as behavior, tonic immobility, feather conditions, presence of body lesions, and antioxidant and immune status: the ultimate aim was therefore to define an index of adaptability to ERS. To this aim, one hundred chickens for each breed then described were purchased and subsequently reared under the organic system; in particular, the utilized strains were: SG: Ancona, Leghorn, crossbreed Cornish × Leghorn; MG: Gaina, Robusta maculata, Kabir, Naked Neck; FG: Ross 308. All the considerate variables were measured at the same age, and birds were slaughtered according to the organic rule (889/2008) at minimum age of 81 d. The rank of adaptability was calculated, based on the 49 different traits recorded for each bird. In particular, the different scores were used for the sum of the final values, in turn chosen for the calculation and normalization of the final index of adaptability. The Ancona and Livorno strains showed a fast response to the tonic immobility test, varied behaviors, and a greater ability to range. The best plumage condition in all anatomical regions was observed in chicks belonging to Ancona, Livorno, Cornish × Livorno, Gaina, and Robusta maculata genotypes, as well as the absolute absence of footpad and breast blister lesions. The static behavior of Ross 308 and Gaina chickens did not produce a significant oxidative burst, whereas in the Ancona birds the intense movement was correlated to the high oxygen demand. Plasma α-tocopherol, one of the main specific antioxidants of membrane lipids, followed the trend of kinetic and foraging activity, being higher in slow-, intermediate-, medium-, and FG birds. The adaptability index showed the best result for SG strains with intermediate results in medium growth and the worst in FG ones (Table 2).

These results demonstrated a negative linear correlation between body growth and ability to adapt to different farming conditions, in contrast to what observed within the same sub-group (slow, medium, and fast), where no correlation between daily weight gain and adaptation to the organic system were observed.

Once again, the Ross 308 chickens showed the highest production performance but again proved unsuitable for ERS. Too high daily weight gain could only be considered a negative prerequisite for chicken adaptation, but it certainly was not enough to choose the most suitable chicken because birds with similar weight gains also showed wide adaptation scores.

In particular, the need was not only to find the most suitable chicken, but also to get birds easily available on the market [17]; for this reason, two large breeder companies were involved in the trials and the relative genotypes were analysed to evaluate their productive performance, behavior, and welfare status under the organic system:

- Aviagen: Ranger Classic (R1), Ranger Gold (R2), Rowan Ranger (R3)
- Hubbard: Hubbard RedJA (A), CY Gen 5 × JA87 (CY), M22 × JA87 (M)

**Figure 4:**
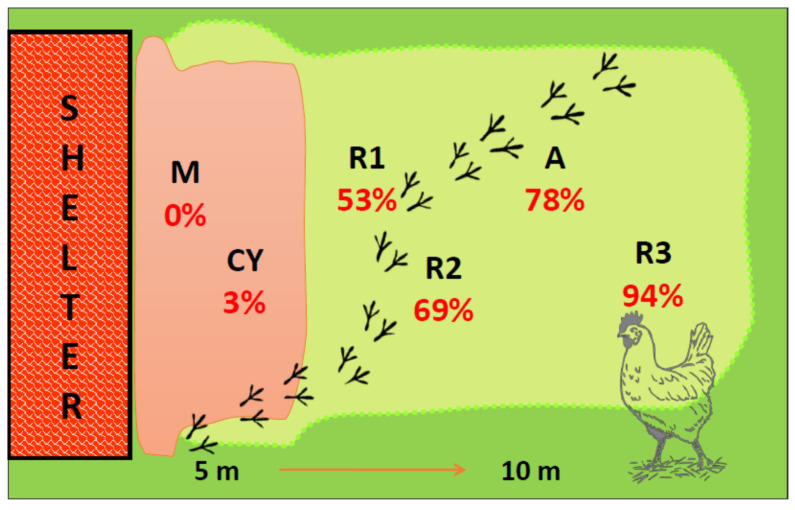
Schematic representation of outdoor run use by different poultry genotypes. (R1) Ranger Classic, (R2) Ranger Gold, (R3) Rowan Ranger, (A) Hubbard RedJA, (CY) Gen 5 × JA87, (M) M22 × JA87. In red percentage of time spent outdoor (respect of total opening time of shelter).

The results showed that A and R3 had good adaptability, showing active behaviors and satisfactory productive performance (final body weights 3083.6 g and 3022.1 g, respectively). Although CY and M achieved the best productive performance, they did not appear adapted to the organic system due to a higher frequency of static behaviors, mortality, footpad dermatitis, breast blisters, and poor feather conditions. Hence, to define the adaptability of poultry genotypes to organic systems, besides the daily weight gain, many other aspects such as behavior, welfare, and health status should be considered to confirm the previous findings. In fact, although R2 and R3 showed a similar daily weight gain, they exhibited a different adaptability, in particular in their use of the outdoor run (Figure 4).

At the conclusion of this set of experiments, it was clear that in ERS, a different (from conventional) equilibrium between performance and adaptability, was necessary to respect the holistic expectation of organic agriculture (animal welfare and health, environment protection, and biodiversity).

## 3. Metabolic Aspects

To better characterize the potential use of the different chicken strains, research was carried out to define further aspects such as health, muscle fiber characteristics, and lipid metabolism; in short, to deepen knowledge and to find better solutions to current problems.

In two studies carried out in collaboration with the Department of Veterinary Medicine at the same university, some aspects relating to intestinal bacterial flora were investigated. In the first one, the intestinal microflora composition in chickens of different rearing systems (conventional vs. organic) and the *Salmonella* prevalence were evaluated [18]. The results showed that the differences between the two groups at the same age, expressed by the bacterial count, are not conclusive in clustering the rearing systems. *Salmonella* was isolated once in caeca of conventional and once in caeca of organic chickens and did not seem to be common in conventional and organic chicken farms.

The major bacteria colonisation of the intestinal tract (*ileum* and *caecum)* of organic and conventional chickens was counted, isolated, and identified by conventional methods [19]. Large differences existed in bacterial counts in relation to the intestinal tract, rearing system, and farms. In the ileum of conventional birds, *Enterobacteria* were higher than in organic birds (7.03 vs. 6.09 CFU × log/g), whereas the opposite was observed for *Lactobacilli* (6.75 vs. 7.07 CFU × log/g). With respect to the other bacteria of microflora, the effect of the farm probably masked possible differences. The effect of rearing system was more visible in the *caecum* than in the *ileum*: *Enterobacteria* levels were higher in conventional than in organic chickens (7.42 vs. 7.05 CFU × log/g), whereas *Enterococci* (7.65 vs. 6.55 CFU × log/g), *Lactobacilli* (7.85 vs. 7.31 CFU × log/g), and total aerobia (8.12 vs. 7.66 CFU × log/g) counts were higher in organic chickens. In both the intestines (*ileum* and *caecum*) of conventional birds, *Enterobacteria* and total aerobia increased with age while *Lactobacilli* decreased. In the organic system, *Enterobacteria*, *Lactobacilli*, and total anaerobia showed a similar trend, whereas total aerobia and *Enterococci* showed the opposite trend. The multivariate analysis (PCA) of the microflora of caecum was able to discriminate the rearing system. Evidently, these can be considered preliminary works, but they have certainly laid the groundwork for further investigations to assess the role and effect of enteric flora on the productive performance and health status of ERS chickens.

**Figure 5:**
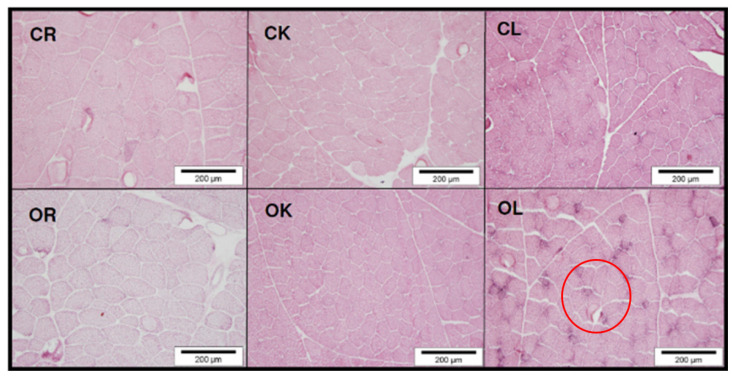
Comparison of *Pectoralis major* muscle cross taken from conventional Ross 308 (CR); conventional Kabir (CK) or conventional Leghorn (CL); and organic Ross 308 (OR), organic Kabir, (OK) or organic Leghorn (OL) chickens; in the circle: α-red fibers (modified by [20]).

In two other investigations, [20,21] also investigated, with researchers of the Department of Veterinary Medicine, the relationships between the type of breeding and poultry breed with related effects on muscle biochemistry, for the first time correlating behavioral traits with myofibrillar characteristics of muscles. Chicks from SG (Leghorn), MG (Kabir), and FG (Ross 308) genotypes were reared according to conventional and organic rearing systems. The behavioral outputs showed that the selection for growth rate modified bird behavior with a significant lowering of kinetic activity. Indeed, Leghorn birds were characterized by high kinetic activities, whereas Kabir and mainly Ross 308 were discriminated on the basis of their lying, standing, and eating activities. These behaviors were strongly associated with energy conservation, growth, and muscle fiber characteristics. The main original finding of this study was that fiber characteristics and the functions of the muscle enzymes are affected by the rearing system only in animals adapted to ERS. Indeed, Leghorn chickens had α-Red fibers in the breast muscle, and the kinetic activity increased the cross-sectional area of the *Ileotibialis lateralis* muscle. These findings, together with behavioral data, confirm that this genotype adapts well to the organic rearing system (Figure 5).

**Figure 6:**
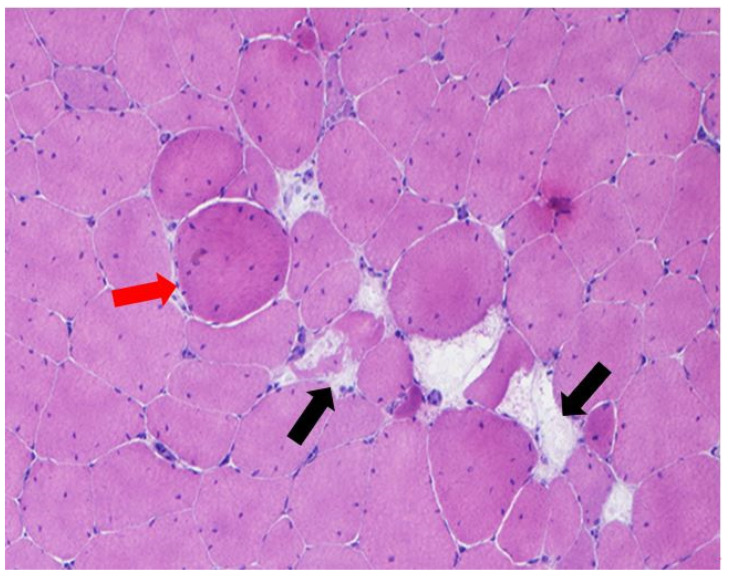
Ross 308 *Pectoralis major* with muscle fibers necrosis (**black arrows**) and giant fiber (**red arrow**). H-E, 200×.

**Figure 7:**
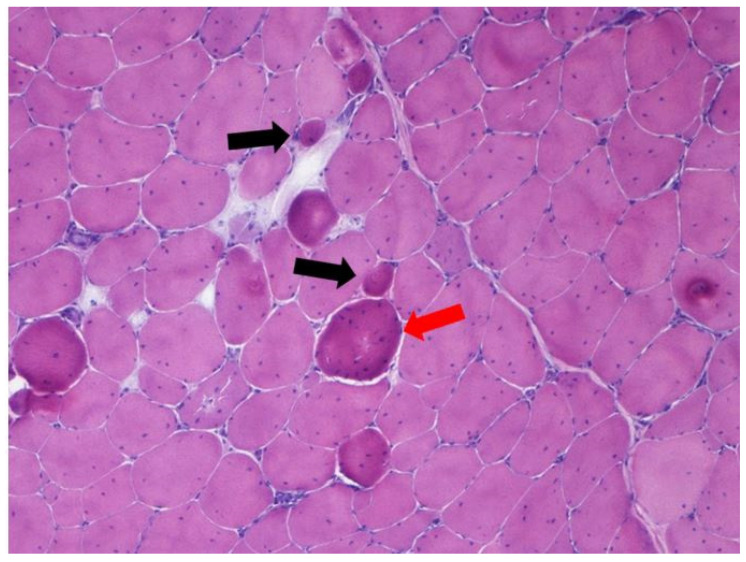
Ross 308 *Pectoralis major*. Presence of hypercontracted atrophic fibers (**black arrows**) and giant fiber (**red arrow**). H-E, 200×.

Moreover, these results indicate that FG chickens are very inactive and that muscle characteristics showed a metabolic and histologic profile less adapted to movement. Indeed, the most current problems around poultry meat quality are associated with deep pectoral muscle disease and white striping, which both impair product appearance, and increased occurrence of problems related to the meat’s reduced ability to hold water during processing and storage as well as poor cohesion related to immaturity of intramuscular connective tissue. In particular, the muscle hypertrophy of these strains may also contribute to the onset and development of breast muscular disorders due to the presence of giant fibers with high cross-sectional area and hypercontracted fibers (HF). Ross 308 chicken samples showed different features of myopathy represented by single-cell necrosis, lymphocytes, plasma cells and macrophage inflammatory infiltrates, fibers regeneration, HF and angulated fibers, fiber splitting, and increased internal nuclei, with similar features to human muscular dystrophies (e.g., Duchenne). On the other hand, only a few Leghorn samples had minor signs of myopathies (Table 3 and Table 4, Figure 6 and Figure 7). Furthermore, the number of Giant Fibers (GF) per field was higher in the FG strain than in the slow- growing one (7.89 vs. 1.77%). The number of capillaries per muscle area was also higher in the SG strain, supporting previous studies that showed a reduced blood supply in breasts of FG strains. Accordingly, breast muscle hypertrophy of meat-type chickens favored fiber dystrophy that may develop muscle anomalies.

Up to this point, the ideas described lead us to think that FG chickens with different ethograms, mainly at an age higher than 40 d, could show locomotor problems, pathological accumulation of fluid in the peritoneal cavity, and other physiological and behavioral anomalies. Under the organic system, with an increase of the locomotor issues, these problems did not decrease but rather became predominant.

In another study [21], the occurrence of GF in muscles with different energy metabolism (*Pectoralis major*, *Iliotibialis lateralis*, and *Semimembranosus*) was studied in Leghorn and Ross 508 chickens. The results showed a small percentage of GF in pre-rigor muscle samples even at 3 min post mortem in both genotypes and in all studied muscles, whereas from 3 min to 24 h post mortem giant fibers increased in both Leghorn and Ross 308 chickens but to different extents according to muscle type and genotype. The highest value of GF, 24 h post mortem, was found in the *Pectoralis major* muscles belonging to the FG line, suggesting that every muscle can develop GF, but this phenomenon is more evident in this muscle, especially in birds selected for high growth rate.

Taken together, these studies showed a reduced blood supply by capillary vessels and a higher presence of GF in fast-growing strains. Moreover, we found different features of myopathy associated with GF. Some represented by HF, splitting fibers, and internal nuclei are also characteristic of human muscular dystrophies. Strong selection pressure for muscle hypertrophy in meat-type chickens favoured fiber dystrophy that may develop breast muscle anomalies, such as white stripping and wooden breast responsible for significant economic losses.

A further metabolic study [22] was carried out in order to evaluate the effect a moderate movement could have on the in vivo oxidative status in different poultry breeds (FG, Ross 38 and SG, Hubbard). Each strain was subjected to different treatments: no exercise and 1 h/day of induced walking at 4 km/h for 4 wks. In both genotypes, the creatine kinase amount (index of muscle damage) was greater in birds that moved more; however, a significantly higher increase was observed in the FG birds, and the antioxidant status was also worse. In contrast, in SG chickens of the experimental group, the antioxidant status remained nearly the same during the experimental period. The α-tocopherol and retinol concentrations decreased with exercise, primarily in the FG group, whereas the other antioxidant compounds (α-T3, γ-T3, γ-T, δ-T, lutein, and zeaxanthin) were unaffected by strain or treatment. The more interesting finding was that the selected strains had different reactions to exercise, and only the SG strains had a linear reduction in TBARS and ROS over the 28-day experiment. Then, a low intensity movement could be considered beneficial only when the chickens show optimal behavioral patterns (e.g., higher kinetic activity, rusticity, and explorative nature) muscular (reduces pain behaviors after inflammatory, non-inflammatory, and neuropathic injury), or body characteristics (low body weight) for organic conditions; on the contrary, high exercise is stressful and negatively influences muscle physiology and wellbeing.

Continuing the assessment of the antioxidant status in ERS chickens, a study was carried out to verify the capacity of organic birds to transfer of bioactive compounds from the pasture to their muscles and to study the antioxidant effect of these molecules on the oxidative processes of the meat [23]. At 21 d of age, Naked Neck cockerels were divided into two homogeneous groups: an indoor group and an organic one (0.12 m^2^/bird indoor and 10 m^2^/bird of grass range, respectively). The higher antioxidant intake of organic chickens resulted in a higher plasma concentration of antioxidants and antioxidant capacity with lower TBARS. The meat of the outdoor birds also showed a significantly higher number of tocopherols and tocotrienols, but notwithstanding this good antioxidant shield protection, the drumstick of the organic group showed a high intensification of oxidative processes, probably due to the movement of chickens, the higher amount of PUFA, and the very high peroxidability index.

Two papers pointed out the effect of slaughtering age (70 and 81 days) in different commercial chicken strains (Naked Neck, Kabir, and Ross 308) organically reared [24]. Naked Neck birds showed the best plumage conditions at both ages; the other genotypes had similar body conditions, showing a dramatic worsening at the end of cycle (81 days). The carcass conformation showed differences mainly for the Naked Neck, which was slenderer with higher proportions of head, neck, and legs; thus, ready-to-cook-carcass yield was lower. The meat of Naked Neck showed lower levels of lipids, pH, and brightness values but higher redness. The Ross 308 genotype showed a poor welfare status even at 70 days (severe foot-pad lesions and breast-blister score, worst feather conditions, and tonic immobility), confirming that the rearing of this strain should be avoided in organic systems. Concerning the content of saturated fatty acids [25], the highest value was observed in Ross 308 chicks. The Naked Neck birds showed the lowest value of saturated fatty acids, whereas Kabir chickens showed intermediate values. The PUFA showed a different trend at both slaughter ages. At 71 days, medium growing (MG) chickens had lower PUFA, while at 81 days Naked Neck birds reached the highest PUFA. The same strain exhibited lower concentrations of linolenic acid but higher long-chain PUFAs. However, the meat of these chickens showed lower lipid stability despite a higher antioxidant content, probably due to the kinetic behavior and the resulting high oxidative metabolism. This finding is of importance because health concerns over fatty acid profiles are among the main risk factors contributing to the reduction of meat intake by humans.

In conclusion, this study indicates that genotype deeply affects the performance, welfare, and qualitative characteristics of meat. Regarding the slaughtering age, although the European rules that authorize the reduction of slaughtering age in SG strains at 70 days are inconsistent, chickens showed higher feed efficiency and leanness of carcass and meat. The results of this trial demonstrate that at an older age, chickens show a better fatty acid profile under a nutritional point of view, even if the oxidative status worsens.

**Figure 8:**
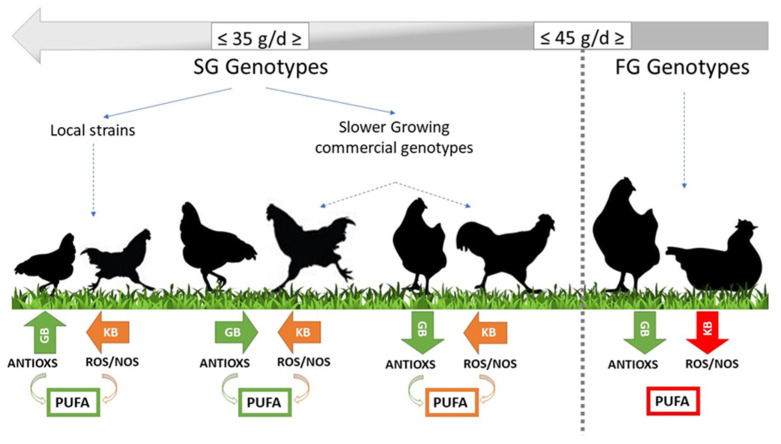
Main oxidative interactions in poultry strains adapted/non-adapted to ERS. GB: grazing behavior, KB: kinetic behavior, ROS: reactive oxygen species, and NOS: reactive nitrogen species PUFA: polyunsaturated fatty acids.

By evaluating these previous studies together, it is possible to have a more definite view of how the genetic strain interacts with movement, intake of antioxidants, antioxidant capacity of the body, plasma, and fatty acid profile of meat. A schematic representation of the main interactions in adapted and non-adapted strains is shown in Figure 8. Once again, the figure reaffirmed that FG chickens are not utilizable in ERS; SG ones generally have a high kinetic behavior that may be matched with a more exploratory attitude. Discrepancy between kinetic and exploratory attitude of SG strains probably explains the differences in the body response of the different strains. In this scenario, from the perspective of improving the meat nutritional characteristics (n-3 PUFA and the n-6 and n-3 ratio), the role of pasture intake (rich in PUFA and antioxidants), which interact with bird activity, must be better understood.

Particular insights were made in two further studies concerning the metabolism of fatty acids and the pathways regulating their anabolism. Indeed, since chickens adapted to ERS are expected to eat a substantial amount of pasture that could modify the fatty acid profiles of their meat, it was necessary to understand the capacity of the body to elongate and desaturate the linolenic acid (ALA, C18:3n-3) of the pasture into long-chain PUFAs such as eicosapentaenoic (EPA, C20:5n-3), docosapentanoic (DPA, C22:5n-3), and docosahexaenoic (DHA, C22:6n-3) acid. The elongation and desaturation ability (generally estimated with Δ6 desaturase activity, the limiting enzyme of the PUFA metabolism) of animals is a very popular topic because, with respect to the world requirement of EPA and DHA, there is an estimated shortage of about 900 t/year [26] that fish and aquaculture are not able to cover.

Accordingly, finding terrestrial and sustainable sources of long chain PUFA is a main goal. From this point of view, the effect of poultry genotype on fatty acid composition and indices of lipid metabolism was evaluated [27] in six poultry genotypes with different growth rates (SG: Leghorn, Ancona, Cornish × Leghorn; MG: Kabir, Naked Neck; FG: Ross 308) that were organically reared. Breast meat fatness, fatty acid composition, and metabolic indices were largely related to genotype, as SG strains had higher elongase, thioesterase, and Δ5/Δ6 desaturase indices accompanied by a lower Δ-9 desaturase (Table 5). 

In summary, the different lipid metabolisms, and the higher efficiency of EPA and DHA deposition in SG animals, could be explained by considering:

1. A specific gene determinism (FADS1 and 2 genes) involved in metabolism of long chain n-3 and n-6 PUFA (see the following study).
2. A different intake of pasture, which increased ALA, antioxidants, phytoestrogens, and polyphenols.

FG strains, selected for meat traits, had a different hormonal profile, whereas the SG strains used in this study were egg-type lines, which probably have a higher efficiency in EPA and DHA deposition than meat-type chickens, as elongation is also affected by oestrogen levels. Finally, as demonstrated so far, SG birds showed higher kinetic activities (walking, running, foraging, exploring, and crouching at pasture) that produced different characteristics and metabolism of muscle fibers. However, further studies are needed to confirm these hypotheses through the direct measurement of enzymatic activities and gene expression of the aforementioned complex.

For this reason, we measured the liver mitochondria of different chicken strains [28]:

- The expression of the desaturating enzymes (Δ6-, Δ5-, and Δ9-desaturase);
- The impact of the different expression on the meat fatty acid composition;
- The distribution of desaturase metabolites in the different lipid classes.

**Figure 9:**
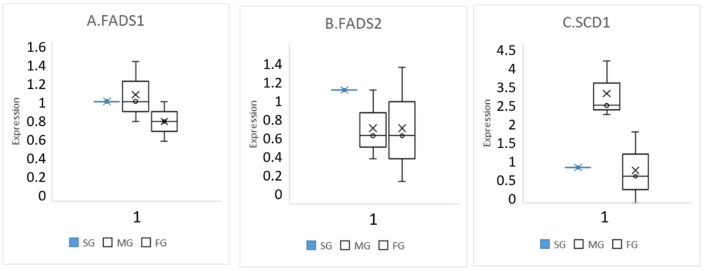
Expression of the genes encoding for the desaturating enzymes in the liver of the three chicken genotypes. (**A**) FADS1, (**B**) FADS2, and (**C**) SCD1 gene expression is reported in the corresponding panel. Data are expressed as median ± range (minimum to maximum). SG: slow growing; MG: medium-growing; and FG: fast growing. Modified by [28].

SG, MG, and FG chickens were evaluated for the relative expression of FADS1, FADS2, and SCD1 genes and for the fatty acid composition of breast meat. The original results confirmed that MG and particularly SG birds showed a greater expression of FADS2 and FADS1 genes, a higher Δ6- and Δ5-desaturase activity, and consequently a higher long-chain PUFA content in the breast meat compared to the FG ones (Figure 9).

The relationship between genotype and desaturating ability was hence clearly demonstrated, with a significant impact on the n-3 PUFA of breast meat.

## 4. Feeding

Feeding represents the greatest cost of production of poultry (about 60–70%), thus reducing this impact by the way of pasture management, and improvement of the characteristics described up to this point is especially important.

Two reviews, written in collaboration with Greek and English researchers [29,30], investigated the various implications of the term “grazing poultry” and defined the different production systems for grazing poultry. Great attention was paid to the welfare of poultry reared in ERS, focusing on potential problems correlated with strain, range and grass. Furthermore, different sustainability precincts were analyzed in order to evaluate the environmental, economic, and social benefits promoted by ERS, considering access to the outdoors as a distinctive feature of this type of production as it is capable of providing fresh grass, insects, and worms that can lead to better product quality and lower production costs. For example, the presence of pasture and consequently the grass ingestion could reduce the feed consumption up to 30% as compared to the same genotypes without outdoor access. There is evidence that grazing poultry meat may have some nutritional benefits such as lower fat, as well as a higher vitamin and mineral content. At the same time, with good pasture management, bird health and well-being can be achieved. In summary, these two reviews provide an overview of pasture management practices that can be employed to prevent potential risks in ERS, such as uncontrolled weather conditions or predator attacks. These studies discuss the various effects of pasture management on poultry health and welfare, including physical comfort, absence of hunger and disease, possibilities for motivated behavior, and meat quality, including consumer and nutritional quality and sensory attributes related to pasture intake.

**Figure 10:**
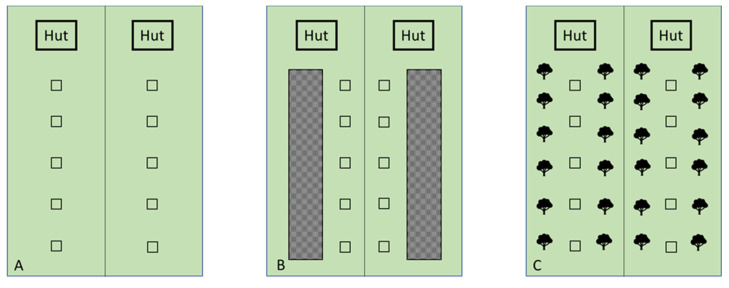
Schematic representation of the different rearing systems: (**A**) = no enrichment; (**B**) = sorghum; (**C**) = olive trees. (Each pen measured 1000 m² each exclusion pen). Modified by [31].

After these extensive reviews, the question that remained was: “How to maximize the use of grazing available to chickens?” In ERS, outdoor runs are not always well managed from an environmental point of view, the pasture is scarcely taken care of (no grass and no shadow point presence) and the chickens’ living space is unsafe. Several shelters should be made available to birds to make the runs more attractive. Considering that the wild ancestors of chickens were preyed on by raptors, modern poultry breeds still instinctively recognize the atavistic danger. Providing bushes and trees inside the pens could help chickens to feel safer from predators and more sheltered from the sun and bad weather; this would allow them to move further away from huts and eat and forage longer. On the basis of these considerations, a trial was carried out to investigate the relationship between range enrichment (trees or tall grass stand), grass intake, and performance of free-range chickens [31] in different seasons (winter and summer). Accordingly, a normal free-range system (grass only) was compared with two range enrichments characterized by *Sorghum* (as a tall grass) and olive trees, respectively (Figure 10). In this experiment, the consumption of grass was also estimated for the first time in meat-type chickens (previously it was only done with laying hens).

Although poultry growth performance was not affected by the different systems, the presence of trees, bushes, or tall grass prevented predation, while, in the standard paddock, cases of predation by raptors or crows were recorded, especially in young chicks. Consequently, a low mortality was observed in the experimental groups. Control chickens stayed more indoors rather than grazing, while in enriched environments, birds spent more time outdoors and made extensive use of available pasture. Estimated forage intake was influenced by farming system and season; the chickens reared in the pens enriched with olive trees showed a greater grass intake, taking advantage of the full grassy area up to almost 50 m from the hut (Table 6).

Along with olive trees, chickens also showed the lowest frequency of footpad and breast lesions.

Other experimental studies, even if not specifically carried out on chickens, can contribute to increasing knowledge on the possible uses of poultry in association with other agricultural productions; in particular, some trials, carried out with laying hens or geese, highlighted the importance of the interaction of the animal and the environment in the well-being and quality of products. Different studies [32,33,34,35] examined the effect of husbandry systems (control, organic, and organic plus, consisting of the use of local breeds with 10 m^2^ pasture/head) and season on the grass intake and egg quality in laying hens. The results showed that grass intake was largely affected by the husbandry system and highlighted the seasonal effect of grass availability on the nutritional quality of eggs. Due to the content of bioactive compounds and n-3 PUFA, the organic plus eggs can be considered functional eggs (>300 mg/100 g egg—Recommendation of EFSA Q-2004-107 for defining an egg as functional), without any dietary supplement.

The agroforestry system could be considered dynamic management of natural resources based on the integration of trees with crops or livestock, which improve natural behavior of animals (that, as prey, find themselves better exploiting the available space and feeling more protected from predators, mainly from birds of prey) and at the same time reduce the land necessary for pasture and provides weed control and manure to the grass and plants. In some studies, we evaluated the grass intake and oxidative status of geese meat reared in different agroforestry systems, e.g., apple orchard, olive trees, and vineyards [34,35]. The results showed that the grazing activity of geese improved the n-3 PUFA, the n-6/n-3 ratio, and the antioxidant content of meat, especially in geese kept in environments enriched with trees. Indeed, the presence of trees protects animals and stimulates them to explore the pasture and consequently consume more grass. However, as revealed in previous papers, the high antioxidant intake through grass was not able to counteract the higher oxidative thrust. Consequently, the meat of these geese was characterized by the worst oxidative status, compared to that of conventionally reared geese.

Another problem that was addressed was the choice of protein sources for organic chicken feed; indeed, synthetic forms of amino acids and genetically modified organism (GMO) ingredients are banned from organic diets. Possible alternatives for organic poultry include the use of SG genotypes that have lower protein and amino acid requirements (especially during the starter phase), and the feed formulation, which utilizes lesser amounts of soybean (at high risk of GMO contamination). One of the most interesting legumes alternative to soybean is the faba bean (*Vicia faba* var. minor), which has received considerable interest as an indigenous source of protein for Mediterranean countries since it can grow in dry areas.

Two papers were published on the effect of faba bean as a partial substitute for soybean based on the performance, carcass traits, and meat quality of organically reared SG chickens (Gaina strain) of both sexes [36,37]. The two experiments were repeated in two seasons (spring and autumn); the difference between the two experiments consisted of the administration of faba only in the grower diets (>21 d starter diet) and also in the starter (until 20 days of life). Subsequently, two different grower-finisher diets containing soybean (24%) or faba bean (16%) until the end of the rearing period (120 days) were used. In the first experiment, the final live weight, feed intake, and daily weight gain were influenced by sex and diet. In particular, the faba dietary plan reduced the productive performance from 21–60 days of age. Subsequently, compensatory growth eliminated the differences in slaughter weight. Chickens fed a growing-finisher diet containing faba bean as a partial substitute for soybean that, after a transition period, showed compensatory growth in the subsequent period, reaching a final live weight (120 d) similar to the soybean group. Thus, the use of faba bean diet without synthetic amino acids, if administered after an initial period, is able to meet the needs of SG birds.

Subsequently, in a second experiment, fava beans were used in both starter (1–21 d) and growth-finisher (22–120 d) diets. It was observed that the performance of the experimental group was the worst, with a significantly higher mortality rate due to a poor body development in the early stages, indicating that faba bean diets are unable to cover the nutritional needs of birds, mainly at the begin of the productive cycle. Hence, under the experimental conditions of the study, the dietary needs of SG chickens are not completely met with a faba bean diet, considering that the content of anti-nutritional compounds probably reduces the digestibility of proteins and consequently the use of amino acids such as lysine, methionine, and cysteine. Chickens fed the faba bean diet showed growth depression, especially in the early rearing period when the gastrointestinal tract was not fully developed and birds were more susceptible to anti-nutritional factors. The quality of the meat was only partially influenced by the dietary treatment with a lower content of PUFA and antioxidants in the fava group, although the oxidative stability showed no differences. The researchers concluded that further tests should have been carried out with dehulled and micronized beans; alternatively, broad bean varieties free of tannins and low in vicine and convicine should be selected.

In another study [38], the effects of partial substitution of soybean with faba bean in different genotypes (FG, SG, and MG) on the lipid composition and meat quality attributes of chickens reared under organic conditions were evaluated. In this study, the partial replacement of soybean with faba bean had a lesser effect than the genotype on the meat quality characteristics.

## 5. Environmental

**Figure 11:**
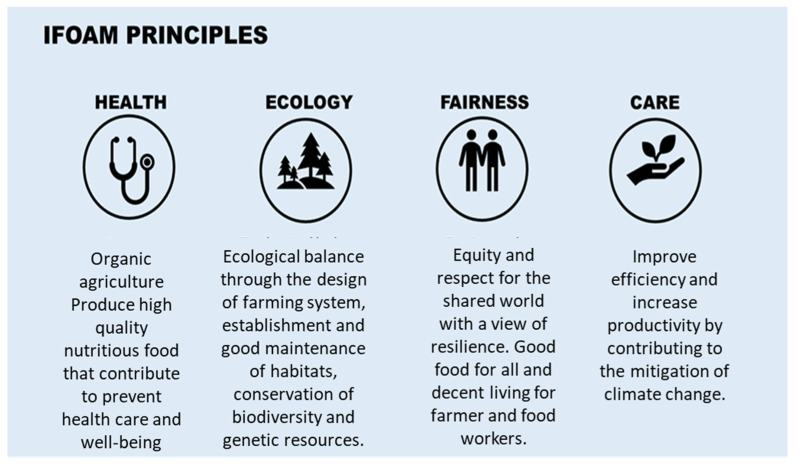
International Federation of Organic Agriculture Movements principles (modified by https://www.ifoam.bio/why-organic/shaping-agriculture/four-principles-organic, accessed on 27 December 2020).

The reduction of the discrepancy between economic performance and the safeguard of the environment was an important pillar of the organic rearing system, where the holistic approach provides a positive impact on different aspects (see International Federation of Organic Agriculture Movements, IFOAM, principles in Figure 11), with the environment being one of the more relevant of the aspects.

Organic agriculture creates circular and closed systems, which are less productive in terms of livestock yields. However, the reduced yields are generally matched by greater energy efficiencies mainly when the comparisons are made on a unit of area basis. Within livestock production, the organic poultry production system (measured on kg product) performs worse where poor feed conversion can lead to lower energy efficiency. Thus, to make a sound comparison of intensive vs. organic systems, the type of resources (type of land used, % of renewable energy) should be considered.

The organic production system avoids the use of synthetic chemical compounds, limiting the intensity of production and providing controls along the entire chain of production. Moreover, the organic system improves local sustainability, whereas the effect on global sustainability is not easily assessable. For this, several methods that consider a certain number of factors and indicators (soil erosion, CO_2_ emission, water pollution, land use, etc.) are available for evaluating the environmental impact of animal production.

One of the first studies in the world on the environmental impact of organic poultry production was published in 2006 [39] using an *Emergy* approach (112 citations, source Scopus). *Emergy*, defined as the solar (equivalent) energy required to generate that flow or storage evaluation, deals at best with systems at the interface between the “natural” and the “human” spheres, and because it is able to account for all inputs on a common basis, it is able to avoid difficulties and subjectivity that could take place with other methods (i.e., Life Cycle Assessment, reviewed by [40,41,42,43,44]). Indeed, traditional energy analysis provides short-term feasibility for a process, but it should be emphasized that all forms of energy do not have the same “quality”, while for other sectors, environmental sustainability using *Emergy* evaluation is widely diffused and information on the impact of different animal production systems is scarce. Hence, the aim of another study [44] was to compare conventional and organic poultry production systems in terms of an *Emergy* analysis. The main differences between the two systems were the *Emergy* cost for poultry feed and for cleaning/sanitization of the buildings between successive productive cycles. The feed always represented more than 50% of the *Emergy* flow. Regarding the agronomic phase, it was shown that using almost all the organic crops, avoiding the use of chemical fertilizers and pesticides saved around 60% *Emergy*. Relating the *Emergy* results with productive performance, it is possible to show that, although the annual productive performance was much lower in organic systems than in conventional (−206%), the transformation of organic poultry was approximately 10% lower. Hence, the comparison of an experimental organic poultry farm with a conventional farm from the viewpoint of sustainability showed that all *Emergy*-based indicators are in favour of organic farming. In particular, *Emergy* showed a higher efficiency in transforming the available inputs in the final product, as well as the level of renewable inputs and local inputs. In contrast, a lower density of energy and matter flows was detected. Nowadays, media and public opinion consider livestock production to be one of the major causes of environmental problems, including global warming, and air and water pollution. Thus, with another study the holistic sustainability of different poultry production systems was analyzed using a multicriteria model to address the quality of food, environmental preservation, economic feasibility, and quality of life. The sustainability of the three poultry production systems (conventional, organic, and organic plus (see previous details)) was compared. A bio-economic model combining direct and indirect traits involved in organic rearing systems was assessed. To create a whole sustainability evaluation, the following four dimensions were considered: economic, social, environmental, and quality. The majority of the data was collected directly on the farms, and the environmental indicators were estimated with a life cycle assessment, ecological footprints, and *Emergy* analysis. To develop the model, six indicators for each dimension (economic, social, qualitative, and environmental) were selected. The analyzed farming systems showed different results based on the stakeholder being considered (scientists, consumers, and producers). The organic plus system showed the best performance when economic, social, and environmental dimensions were integrated following the scientist and consumer stakeholders’ criteria. To our knowledge, this is the first study to evaluate the effect of a poultry farming system by developing a method that summarized different aspects of the production chain. This approach was more holistic because there was no homology of farming systems in terms of animal welfare, product characteristics, landscape aesthetics, and biodiversity.

## 6. Logistical

Another important line of research was aimed at defining various logistical aspects of the ERS supply chain, always preserving animal welfare, the hygienic, sanitary, and qualitative characteristics of the production. In particular, some trials were carried out in order to better define the effect of slaughtering age, transport length, and slaughtering modalities.

Some of our papers indicated [45,46] that the SG strains adopted in ERS could have a different response to the slaughtering process and thus different meat products would result. In this view, two other studies analyzed the effect of transport on welfare traits, several haematological parameters, in vivo oxidative status, carcase hygiene, and breast meat characteristics in two different chicken genotypes (FG and SG strains) reared under free-range conditions. For this aim, cockerels of FG (Ross 308) and SG (Naked Neck) strains were farmed. At the end of the rearing period, at 81 days of age, chickens were randomly selected for slaughter and subjected to two different pre-slaughter conditions: no transport (0 h) or 4 h of transport (4 h). The transport length significantly affected the in vivo oxidative status of chickens, greatly reducing the α- and δ-tocopherol, retinol, lutein, and zeaxanthin content in plasma, and increased oxidative stress in both strains. Cholesterol and triglycerides were not different between the experimental groups, whereas glucose decreased after 4 h of transport in either strain. A significant difference between groups for the heterophil/lymphocyte ratio after transport was also observed, with Naked Neck being higher than that of Ross 308. Concerning oxidative stress, we observed higher reactive oxygen species (ROS) production in Naked Neck birds. In both strains (Ross 308 and NN), the carcass microbial characteristics showed a higher level of contamination after 4 h of transport (total viable counts) but not with *Enterobacteriaceae* counts. The pH (0, 2, and 24 h post-mortem) of breast muscles of chickens transported for 4 h showed higher values, and with respect to strains, Naked Neck had lower values. In all the stains, the pH values were negatively correlated with lightness (2–24 h) and shear force of meat. The transport length also affected the fatty acid profile of breast muscle, with a decrease in PUFA and an increase in oxidative processes. The antioxidant content of the breast was reduced by chicken transport (α-tocotrienol, α-, δ-tocopherol, lutein, and zeaxanthin), especially in Naked Neck birds. In conclusion, the results indicate that transport for 4 h prior to slaughter negatively affects the meat quality of poultry mainly in SG chickens, which seem more sensitive to stress transport due to the higher kinetic behavior of these strains.

**Figure 12:**
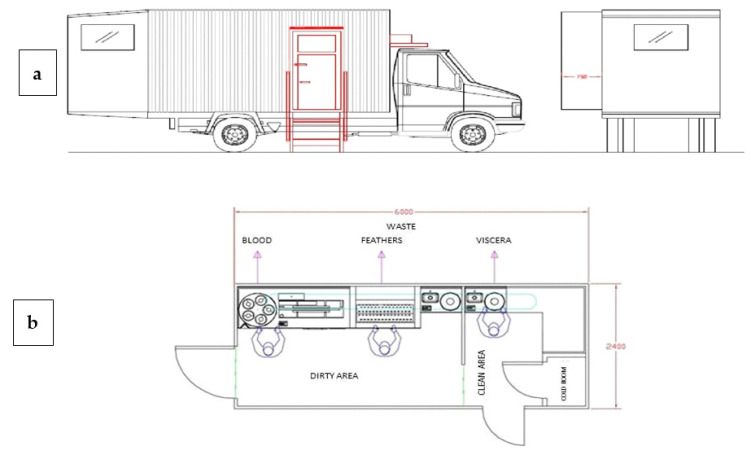
Schematic layout of the Mobile Poultry Processing Unit (50 chickens/hour): external (**a**) and internal (**b**) configuration. Modified by [47].

Another major criticism of ERS is the slaughtering house. Indeed, on the one hand, small and medium farms have difficulties in accessing private slaughtering houses, and on the other hand, we have just seen that SG chickens suffer from long trips to slaughtering houses. A possible solution could be represented by the use of a mobile poultry processing unit (Figure 12) that travels directly to the poultry farms.

Thus, the University of Perugia and other private partners planned and developed a mobile poultry unit [47], characterized by the electric stunned and a work capability of 50 chickens/h. In this paper, the economic efficiency, animal welfare aspects, and the qualitative and sanitary implications were analyzed.

Qualitative and societal aspects are discussed together with bird welfare and hygiene implications. The analysis of the case study indicates the viability of mobile poultry processing units but at the same time notes that up-scaling to medium sized operations would not be permissible under current EU regulations. Despite the improvement in poultry welfare (no transport or limited period of transport), the geographical and numerical limitations imposed by EU regulations mean that prototype development for medium- or large-scale poultry production is unlikely to occur. European legislation has limited further improvement (e.g., different stunning systems, carcass decontamination, and water-bath chilling), confining ERS production to local production and selling. Nonetheless, other countries could take advantage of this experience and improve this prototype according to national legislation. In developing countries where the demand for livestock products is strongly increased and in many cases the society is organized in small and poorly connected units, the mobile poultry processing unit could represent a real and feasible opportunity of progress.

## 7. Technological

During these years of study, the evidence of the findings led us to consider quality not an instant fact but, on the contrary, the result of a long process that starts from the genetic project and ends in the consumer’s dish: “in short, not a picture but a movie.”

**Figure 13:**
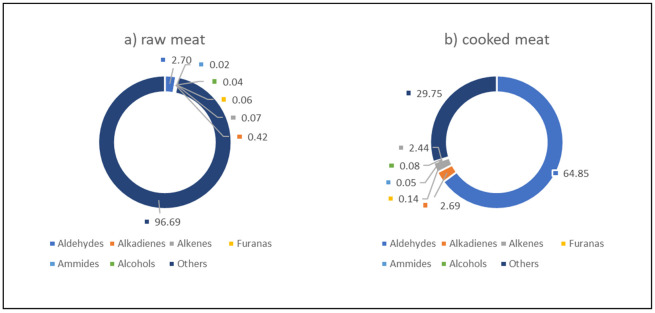
Main VOC classes (%) in raw (**A**) and cooked (**B**) chicken meat. Modified by [47]. VOC: volatile organic compound.

Especially in food such as poultry, so rich in unsaturated fatty acids, the care during technological processes and particularly in cooking is essential in order to preserve it quality. For this reason, a study [48] was carried out with the aim to evaluate the fatty acids, antioxidants, and volatile organic compound (VOC) profiles of raw and cooked meat. To this aim, samples derived from four strains of chicken, conventionally reared but differing in their growth rates: SG (Livorno), MG (Hubbard and Naked Neck), and FG (Ross 308), were used. The results revealed that the cooking method (Figure 13) and the chicken genotype (Table 7) strongly influenced the VOC profile of the meat. Identifying the relationships between these traits highlighted the trade-off of the main substrates such as n-3 and n-6 PUFA, antioxidants, and VOC of the poultry meat produced during cooking. VOC production and loss of n-3 PUFA during cooking were higher for the SG genotype. Unexpectedly, the reduction of n-6 was higher in MG birds, whereas small losses in antioxidants and PUFA were observed in the FG genotype, resulting in the lowest production of VOCs. The SG and MG genotypes are more active from a kinetic point of view than the FG ones.

**Figure 14:**
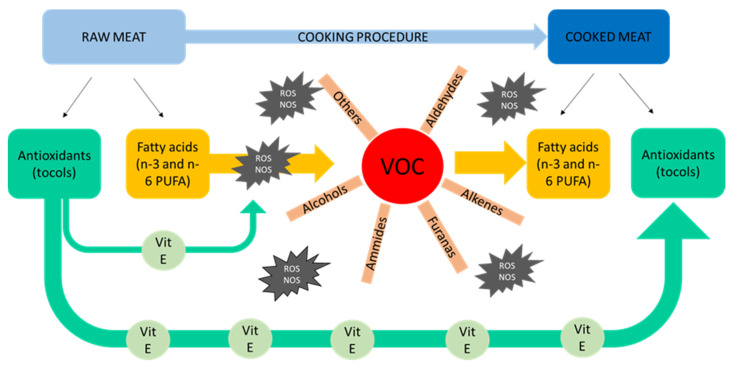
PUFA, VOC, and antioxidant dynamics in poultry meat during cooking process. NOS: reactive nitrogen species; PUFA: polyunsaturated fatty acid; ROS: reactive oxygen species; and VOC: volatile organic compound. Modified by [48].

For this reason, in the FG strains, the antioxidants are less involved in the oxidative stress induced by movement, thus remaining more available to protect the meat fatty acids during the cooking process. These results indicated that SG and MG genotypes require specific dietary protocols (i.e., increasing the antioxidant content) in order to reduce the lipid oxidation in all the phases: in vivo, post-mortem, and during/after cooking (Figure 14). It should be noted that this experiment was executed indoors with no access to the pasture so that all the previous comments on the more exploratory of SG birds did not occur. Accordingly, it could be interesting to perform the same study by the use of these genotypes reared in ERS condition.

## 8. Conclusions

We are concluding this long scientific journey, which started with *number one* and lasted twenty years. The first thing that comes to mind is that it will most likely take many years to identify the right chicken for ERS. Many other multidisciplinary studies are already underway to investigate aspects related to the identification of the genes responsible for the characteristics of adaptability and their expression, from an in-depth study of the relationships between kinetic activity, grazing behavior, antioxidant capacity (blood and meat), and nutritional characteristics of meat; to a study on its economic viability; to a study of other multi-criteria models [43]. Not to be forgotten are the aspects linked to the promotion of ERS products and the development of knowledge relating to the relationships between qualitative characteristics and consumer perception, reviewed in previous papers [49,50,51].

In this context, the aim of the present review was to focus on the relationship between the kinetic behaviors (as a set of different parameters), the carcass characteristics, and the oxidative status of chicken genotypes. Suitable strategies to optimize the link between chick and environment have been defined, and others are being studied. As the following examples show:

- Reduction of kinetic activity in the last period of life;
- Use of new breeds or crossbreeds or, alternatively, new commercial strains selected for ERS adapted to kinetic activity and poor environment conditions;
- Providing more natural antioxidants in the feed or through access to grass outdoors [52,53];
- Avoiding excessive carcass processing and reducing storage time;
- Using cooking techniques that do not affect the unsaturated fatty acids contained in the meat.

Therefore, the challenge of identifying the best genotypes for meat production continues, representing an important goal not only for the food industry but also for the improvement of human nutrition. Only the definition of the best strains adapted to the ERS through the measurement of a wide panel of genetic, physiologic, and behavioral traits and not only relying on daily weight gain will allow us to achieve this goal.

## Figures and Tables

**Table 1:** Global positioning system average outcomes of organic chickens. Modified from [12].

Item	Fast Growing	Slow Growing	Pooled SE
Overall daily distance, m/d	125 ^b^	1230 ^a^	120
Maximum distance from house, m	25 ^b^	100 ^a^	14
Time spent outdoors, % total	25.6 ^b^	74.9 ^a^	25.7
Mean speed, m/h	8.93 ^b^	95.71 ^a^	30.5

^a,b^: Means within rows with different superscripts differ significantly at *p* < 0.05. SE: Standard error.

**Table 2:** Adaptability indexes of different poultry genotype. Modified from [16].

Adaptability index	L	A	CL	G	RM	K	NN	R	Pooled SE
	0.49 ^d^	0.50 ^d^	0.58 ^d^	0.41 ^b^	0.94 ^c^	0.56 ^b^	0.18 ^c^	−1.77 ^a^	0.50
Mean and SD	Slow Growing			Medium growing				Fast growing	
	0.53 ± 0.41			0.05 ± 0.90				−1.77 ± 0.48	

L, Leghorn; A, Ancona; CL, crossbreed Cornish × Leghorn; G, Gaina; RM, Robusta maculata; K, Kabir; NN, Naked Neck, R, Ross 308. (SG < 24 g/d; MG 25 < GR < 40 g/d and FG > 40 g/d). ^a–d^ Values within a row with different superscripts differ significantly at *p* ≤ 0.05.

**Table 3:** Number of fibers per microscopic field and average number of capillaries per microscopic field.

	Ross 308	Leghorn	SEM
Fibers (n/microscopic field)	49.99 ^a^	140.15 ^b^	3.01
Capillaries (n/microscopic field)	24.56 ^b^	22.35 ^a^	0.92

^a,b^ Values within a row with different superscripts differ significantly at *p* ≤ 0.05.

**Table 4:** Average number of giant fibers for different chicken strains.

	Ross 308	Leghorn	SEM
Giant fibers (n/microscopic field)	7.89 ^b^	1.77 ^a^	1.17

^a,b^ Values within a row with different superscripts differ significantly at *p* ≤ 0.05.

**Table 5:** Estimated indices of fatty acid metabolism (on the basis of fatty acid composition) in breast muscle of different genotypes. Modified from [25].

Item	L	A	CL	K	NN	R	Pooled SEM
Elongase	0.46 ^b^	0.47 ^b^	0.37 ^ab^	0.27 ^a^	0.33 ^a^	0.33 c	0.11
Thioesterase	59.8 ^d^	42.7 ^c^	28.3 ^b^	19.4 ^a^	20.3 ^a^	30.9 ^b^	5.33
Δ9-desaturase (18)	55.9 ^a^	58.0 ^a^	62.1 ^a^	71.1 ^b^	68.5 ^a^	68.7 ^c^	7.47
Δ9-desaturase (16 + 18)	29.3 ^a^	31.4 ^a^	32.4 ^a^	37.6 ^b^	34.9 ^b^	37.5 ^b^	4.30
Δ5/Δ6-desaturase (18)	52.5 ^b^	52.4 ^b^	23.6 ^a^	28.0 ^a^	26.4 ^a^	23.5 ^a^	4.75

^a–d^ Values with different superscripts within a column differ at *p* ≤ 0.05. L: Leghorn; A: Ancona; CL: crossbreed Cornish × Leghorn; K: Kabir; NN: Naked Neck; and R: Ross 308.

**Table 6:** Estimated grass intake of chickens (g of dry matter/d per bird). Modified from [31].

	Summer			Winter		
Item	No Enrichment	Sorghum	Olive Trees	No Enrichment	Sorghum	Olive Trees
Distance (m) from the hut						
7	9.00 ^a^	14.23 ^b^	16.70 ^c^	9.15 ^a^	10.12 ^a^	9.92 ^a^
12	4.21 ^a^	8.96 ^b^	12.47 ^c^	3.91 ^a^	4.52 ^a^	7.91 ^b^
17	1.59 ^a^	5.60 ^c^	5.54 ^c^	1.89 ^a^	3.25 ^b^	4.89 ^b^
22	0.00 ^a^	1.57 ^b^	4.19 ^c^	0.00 ^a^	0.00 ^a^	2.54 ^b^
27	0.00 ^a^	0.00 ^a^	2.88 ^c^	0.00 ^a^	0.00 ^a^	1.00 ^b^
47	0.00 ^a^	0.00 ^a^	1.20 ^c^	0.00 ^a^	0.00 ^a^	0.21 ^b^
Total	14.80 ^a^	30.36 ^b^	42.98 ^c^	14.95 ^a^	17.89 ^a^	26.47 ^b^

^a–c^ Means within rows having different superscripts differ significantly at *p ≤* 0.05.

**Table 7:** Influence of genetic strains and processing (raw vs. cooked) on the VOC production (ppb), fatty acid (mg/g DM), and antioxidants (g/g DM) content of poultry meat. Modified by [47].

Compounds	RAW				COOKED				Pooled SE
Genotypes	LEG	HUB	NN	ROSS	LEG	HUB	NN	ROSS	
Total VOC	244.1	2477.3	2550.9	1771.3	11,487.8	8775.8	9698.7	5389.9	3210.0
Σ n-6 PUFA	3.60 ^a^	7.30 ^b^	6.00 ^b^	6.80 ^b^	3.08 ^a^	5.38 ^b^	4.43 ^ab^	6.09 ^b^	1.38
Σ n-3 PUFA	0.78 ^a^	1.14 ^b^	0.82 ^a^	1.03 ^b^	0.44 ^a^	0.79 ^b^	0.63 ^ab^	0.82 ^b^	0.13
Σ-T	19.50 ^a^	41.76 ^c^	28.22 ^b^	42.19 ^c^	1.28 ^a^	5.80 ^b^	4.20 ^b^	12.04 ^c^	1.82

VOC: volatile organic compounds; PUFA: polyunsaturated fatty acids; T: tocols; HUB: Hubbard; NN: Naked Neck; LEG: Leghorn; ROSS: Ross 308; SE: standard error; ^a–c^ Means within rows bearing different superscripts differ significantly at *p* < 0.05.

## Data Availability

Not applicable.

## Supplementary Materials

The following are available online at https://www.mdpi.com/article/10.3390/ani11051281/s1, Table S1: Principal characteristic of the studied genotypes.

## Abbreviations

ERS	extensive rearing systems
PUFA	polyunsaturated fatty acids
TBARS	thiobarbituric reactive substances
SNPs	single nucleotide polymorphisms
FG	fast growing
SG	slow growing
HF	hypercontracted fibers
GF	giant fibers
MG	medium-growing
GMO	genetically modified organism
ROS	reactive oxygen species
VOC	volatile organic compound
ALA	linolenic acid
EPA	eicosapentaenoic acid
DPA	docosapentanoic acid
DHA	docosahexaenoic acid
