# Supplementary material for: Extensive Rearing Systems in Poultry Production: The Right Chicken for the Right Farming System. A Review of Twenty Years of Scientific Research in Perugia University, Italy
Source: Animals (Basel). 2021 Apr 29;11(5):1281. doi: 10.3390/ani11051281 (PMC8145382; doi:10.3390/ani11051281)
Supplement: Supplementary file 1 [file animals-11-01281-s001.zip › animals-1138015-supplementary.docx]

Table 1. Principal characteristic of the studied genotypes.

| **Genotype Classification** | **Genotypes used** | **Daily Weight Gain (g/d)** |
| --- | --- | --- |
| FG | Ross 308  Cobb 700 | g/d > 45 |
| MG | Kabir  Naked Neck  Brown Classic Lohman  Hubbard | 35 < g/d <45 |
| SG | Robusta maculata  Gaina  Ancona crossbred,  Ancona,  crossbreed Cornish × Leghorn | <  35 g/d |

FG Fast Growing, MG Medium Growing, SG Slow Growing.
